# Membrane Lipidome Reorganization Correlates with the Fate of Neuroblastoma Cells Supplemented with Fatty Acids

**DOI:** 10.1371/journal.pone.0055537

**Published:** 2013-02-06

**Authors:** Andrea Bolognesi, Alexandros Chatgilialoglu, Letizia Polito, Carla Ferreri

**Affiliations:** 1 Dipartimento di Medicina Specialistica, Diagnostica e Sperimentale, “Alma Mater Studiorum” Università di Bologna, Bologna, Italy; 2 Istituto per la Sintesi Organica e la Fotoreattività, Consiglio Nazionale delle Ricerche, Bologna, Italy; IISER-TVM, India

## Abstract

Palmitic acid is known to be apoptotic for nervous cells but no data are available on membrane lipidome transformations occurring during its supplementation, although membrane lipids are clearly involved in the apoptotic signaling cascade. NB100 neuroblastoma cells were supplemented with palmitic acid and membrane fatty acids were isolated, derivatized and analysed by gas chromatography at defined time intervals. Parallely, cell viability, morphology, apoptosis, cPLA_2_ and caspase activations were checked. Interestingly, under 150 µM supplementation the incorporation of palmitic acid was accompanied by the specific release of arachidonic acid. This event was timely correlated with cPLA_2_ and caspases activations, and the time window of 60 minutes was envisaged for crucial membrane lipidome changes. The simultaneous addition of 50 µM oleic, 50 µM arachidonic and 150 µM palmitic acids to the cell cultures influenced membrane changes with suppression of caspase activation and maintenance of cell viability. These results highlight the role of the membrane asset with fatty acid remodeling and suggest the potential of lipid-based strategies for influencing cell response and fate in human diseases, such as neurodegenerative disorders or tumours.

## Introduction

The function and organization of lipids in membranes strongly affect cell structure and functions [1]. Up until the development of modern lipidomic analyses this field of research has developed slowly. The importance of modern lipidomics to medicine is critical since alterations of lipid metabolism and membrane functions are associated with various human diseases [2]. Lipidomics has standing out as a research area with diverse goals, from mapping the entire spectrum of lipids in organisms to describing the function and metabolism of individual lipids. This is also connected to the emerging roles of lipids as signaling molecules [3]–[5].

Membrane lipidomics evaluates the composition of phospholipids and in particular follows up their dynamical changes under diverse metabolic conditions, focusing on the type and quantity of fatty acid residues, which crucially regulate membrane structure and functions [6], [7]. The homeostatic nature of these interactions, that govern the biophysical properties of membranes, connects the choice of membrane components with biochemical pathways, and this signaling network is not well understood [8]. Fatty acid analysis can be obtained by ordinary steps of membrane lipid isolation and extraction, derivatization to methyl esters (FAME) and characterization by gas chromatography [9].

In this context, unsaturated fatty acids can be studied also for their geometrical configuration, since the naturally occurring membrane fatty acids have the cis geometry provided by desaturase enzymes, whereas the trans isomers can be formed only in bacteria [10]. We have been involved in the recent years in the study of cellular stress, focusing on the role of cis to trans conversion of unsaturated lipid configuration that is a marker of free radical stress [11]–[13]. Moreover, we have been interested in the role of membrane fatty acid components inducing also favorable effects, such as the monounsaturated fatty acids as markers of longevity found in the erythrocytes from centenarian offspring [14].

Palmitic acid has attracted our attention since it is described to have contrasting effects in cells, such as inducing cell growth, on one hand, [15] or apoptosis on the other [16]–[21]. Moreover, saturated fatty acids and arachidonic acid have been implicated in the molecular mechanisms underlying apoptotic death of nervous cells following ischemic and traumatic events [22]–[25], as well as of other cells [26]–[29]. The activation of apoptosis in these cells has been associated with a massive liberation of these saturated fatty acid and arachidonic acid. These important investigations lack, however, on changes in the membrane fatty acid composition, due to lipid turnover, which is induced either by the supplementation or by the free fatty acid liberation. Indeed, membranes are not inert structures and changes in their composition and lipid assembly may affect profoundly cell structure and functions.

In the present study, we aimed at analyzing the fatty acid composition of human neuroblastoma cell membranes (NB100) under palmitic acid (16∶0, PA) supplementation at different concentrations. In parallel the morphology, viability, apoptosis (caspase activation) and the pathway of cytosolic phospholipase A_2_ (cPLA_2_) [22]–[25] were determined. We expected that the results of lipidome analysis would highlight the central role of the palmitic acid-induced arachidonic acid release from membranes on cell survival. Moreover, we anticipate that this approach was useful to design tailored experiments of fatty acid supplementation that counteract the apoptotic effects due to the induced alterations in membrane lipid composition.

In general, this study intends to emphasize the benefits of application of lipidome monitoring during lipid supplementation, in order to follow-up the fatty acid status and changes influencing membrane reorganization in synergy with the study of biochemical cascades.

## Materials and Methods

### Reagents and Instruments

RPMI 1640, Fetal Calf Serum (FCS), L-Glutamine, antibiotics, trypan blue, fatty acids, n-hexane, chloroform and methanol were purchased from Sigma-Aldrich, San Louis, MO. Flasks and plates were from Falcon, BD Biosciences, NJ. Trypsin/EDTA was from BioWhittaker Europe, Verviers, Belgium. CellTiter 96 Aqueous One Solution Cell Proliferation Assay, Caspase luminescent assays were from Promega Corporation, Madison, WI. DAPI-Antifade was from Resnova SRL, Genzano di Roma, Italy. Other reagents used were from Sigma-Aldrich and Carlo Erba, Milano, Italy.

Viability was evaluated by measuring absorbance at 490 nm by a microtiter plate reader Multiskan EX (ThermoLabSystems, Basingstoke, UK). Phase contrast microscopy was carried out with a Wilovert Standard PH 20 (HUND, Wetzlar, Germany) and a digital camera from Motic Microscopes, China. Fluorescence microscopy was performed with a Nikon Eclipse E600 fluorescence microscope equipped with a Nikon-dedicated digital camera, DXM1200F (Nikon, Tokyo, Japan).

### Phospholipid Extraction and Fatty Acid Analysis

Cells (3×10^6^) were thoroughly washed with phosphate buffer, added with water and pelleted by centrifugation at 14,000×*g* for 40 min at 4°C. The pellet was resuspended in pure water and centrifuged, then, was dissolved in 2∶1 chloroform:methanol and examined by thin layer chromatography (TLC using a bidimensional system; first eluent: chloroform:methanol:acetic acid:water 55∶33:9∶3; second eluent: hexane:diethyl ether:acetic acid 30∶29:1), according to published procedures [22], [26] to determine the purity of the phospholipid fraction. The phospholipid extract was then treated with 0.5 M KOH/MeOH for 10 min at room temperature to convert the fatty acid residues of the phospholipids into their corresponding fatty acid methyl esters (FAME). After this transesterification step, FAMEs were extracted with *n*-hexane, and analyzed by GC. Geometrical trans fatty acids (*trans*-FA) were identified by comparison with standard references obtained by synthesis, as described [12]. Fatty acid compositions are listed in Tables 1, 2, 3 as relative percentages of the total fatty acid content.

**Table 1:** Membrane phospholipid fatty acids of NB100 cells treated for the indicated times with 150 µM PA and compared to controls grown in the same conditions without PA supplementation.

FAME^a^	Control^b^	30′^b^	1 h^b^	1 h30′^b^	2 h^b^	2 h30′^b^	3 h^b^
	(n = 9)	(n = 4)	(n = 4)	(n = 4)	(n = 4)	(n = 4)	(n = 6)
16∶0	26.6±1.8	32.2±0.2^e^	38.7±1.6^e^	40.2±0.3^e^	43.1±0.2^e^	44.8±0.7^e^	45±1.4^c^
9*c*-16∶1	6.7±0.7	5.4±0.1	5.1±0.0^f^	4.7±0.1^f^	4.7±0.0^f^	5.0±0.0^f^	6.4±0.9
18∶0	14.4±1.2	10.1±0.4^f^	9.3±1.4^f^	10.7±0.5^f^	10.2±0.2^f^	8.6±0.6^f^	10.7±1.4^d^
9*t-*18∶1	0.4±0.4	0.1±0.0^f^	0.2±0.0^f^	0.2±0.0^f^	0.2±0.0^f^	0.2±0^f^	0.3±0.2
9*c*-18∶1	32.3±2.0	31.4±0.1	28.3±0.3^f^	26.7±0.1^f^	24.9±0.2^f^	24.6±0.1^f^	22.4±1.4^d^
11*c*-18∶1	8.2±1.0	6.1±0.2^h^	5.3±0.2^f^	5.0±0.1^f^	5.0±0.1^f^	4.3±0.1^f^	5.3±0.7^d^
9*c,*12*c*-18∶2	2.3±0.1	4.4±0.2^e^	4.3±0.4^e^	4.0±0.3^e^	3.4±0.1^e^	5.0±0.5^e^	2.7±1.4
8*c*,11*c*,14*c*-20∶3	0.8±0.4	1.1±0.0	1.3±0.1	1.1±0.0	1.3±0.0	1.4±0.2^g^	0.8±0.4
5*c*, 8*c,*11*c*,14*c*-20∶4	4.2±0.3	5.1±0.1^e^	4.4±0.0	3.7±0.1^f^	3.6±0.1^f^	3.1±0.1^f^	3.4±0.2^d^
*trans*-20∶4*	0.5±0.2	0.1±0.0^f^	0.0±0.0^f^	0.5±0.0	0.4±0.2	0.4±0.1	0.3±0.1^h^
SFA	40.9±3.0	42.3±0.2	48±0.2^e^	50.9±0.2^f^	53.3±0.0^e^	53.4±0.1^e^	55.7±1.9^c^
MUFA	47.3±3.8	43.1±0.1	38.9±0.4^f^	36.6±0.1^f^	34.7±0.1^f^	34.1±0.3^f^	34.3±0.9^d^
PUFA	10.5±0.5	13.9±0.4^e^	12.3±0.4^e^	11.2±0.3^g^	10.6±0.2	11.5±0.3^g^	9.0±0.3^f^
SFA/MUFA	0.9±0.2	1.0±0.0	1.2±0.0^e^	1.4±0.0^e^	1.5±0.0^e^	1.6±0.0^e^	1.6±0.1^c^
Tot. Omega 6	8.2±0.3	10.6±0.4	9.9±0.6	8.9±0.3	8.4±0.1	9.4±0.4	9.0±0.4^c^
Tot. Omega 3#	3.7±1.2	3.3±0.1	2.2±0.2	2.2±0.6	2.1±0.3	2.0±0.4	1.6±0.3^f^

**Controls are the mean of cells cultured from 0 to 3 hours in the absence of PA. The values are reported as % rel of the total fatty acid peak areas detected in the GC analysis. They are mean values ± SD of the n repetitions of the same experiment.**

a FAME are obtained from total lipid extraction, derivatization, and GC analysis.

b The identification of the peaks have been performed by authentic samples and the identified peaks accounted for >98% of the total peaks.

c Values higher than untreated control (***P = 0.0001).

d Values lower than untreated control (***P = 0.0001).

e Values higher than untreated control (**P<0.001).

f Values lower than untreated control (**P<0.001).

g Values higher than untreated control (*P<0.01).

h Values lower than untreated control (*P<0.01).

* Evaluated with standard compounds (mono-trans arachidonic acid isomers) obtained following references [12].

# This value includes EPA and DHA.

**Table 2:** Membrane phospholipid fatty acids of NB100 cells treated for the indicated times with 50 µM and 150 µM PA and compared to controls grown in the same conditions without PA supplementation at each time intervals.

FAME^a^	Control 8 h^b^	50 µM PA 8 h	150 µM PA 8 h	Control 24 h^b^	50 µM PA 24 h	150 µM PA 24 h
	(n = 4)	(n = 4)	(n = 4)	(n = 4)	(n = 4)	(n = 4)
16∶0	24.9±0.3	27.5±0.2^c^	44.0±2.6^c^	26.3±0.6	29.7±0.5^c^	47.2±0.6^c^
9*c*-16∶1	7.3±0.2	7.8±0.2^e^	9.6±0.5^c^	6.7±0.3	9.0±0.3^c^	13.1±0.3^c^
18∶0	13.0±0.3	13.4±0.1^g^	11.5±0.5^f^	14.2±0.8	13.5±0.4	10.0±1.1^d^
9*t-*18∶1	0.2±0.0	0.4±0.1^g^	0.5±0.0^c^	0.3±0.1	0.3±0.0	0.3±0.1
9*c*-18∶1	33.7±0.4	31.2±0.0^d^	20.7±1.6^f^	32.6±0.9	27.2±0.1^d^	15.4±0.1^d^
11*c*-18∶1	8.6±0.3	9.3±0.1^e^	6.5±0.5^d^	8.6±0.3	10.3±0.3^c^	7.7±0.3^f^
9*c,*12*c*-18∶2	2.4±0.08	2.1±0.03^d^	1.6±0.1^d^	2.3±0.2	2.1±0.1	1.8±0.1^f^
8*c*,11*c*,14*c*-20∶3	0.7±0.0	0.7±0.0	0.7±0.1	0.7±0.0	0.6±0.0^f^	0.6±0.0^d^
5*c*, 8*c,*11*c*,14*c*-20∶4	4.8±0.3	4.0±0.1^f^	3.1±0.3^d^	4.4±0.2	3.9±0.1^f^	2.5±0.1^f^
*trans*-20∶4*	0.6±0.2	0.6±0.1	0.2±0.0^f^	0.5±0.1	0.7±0.3	0.3±0.0^h^
SFA	37.9±0.1	40.9±0.2^c^	55.5±3.1^c^	40.5±1.3	43.2±0.9^g^	57.2±1.2^c^
MUFA	49.5±0.3	48.3±0.3^d^	36.7±2.6^d^	47.9±1.4	46.4±0.5	36.1±0.5^d^
PUFA	10.9±0.3	9.4±0.2^d^	7.5±0.5^d^	10.7±0.7	9.3±0.5^h^	6.8±0.1^d^
SFA/MUFA	0.7±0.0	0.8±0.0^c^	1.5±0.2^c^	0.8±0.1	0.6±0.4	1.6±0.0^c^
Tot. Omega 6	8.1±0.3	6.9±0.2^d^	5.4±0.3^d^	7.6±0.3	6.9±0.1^f^	4.9±0.1^d^
Tot. Omega 3#	2.8±0.1	1.9±0.1^d^	1.7±0.1^d^	3.1±0.4	2.4±0.2^h^	1.9±0.0^f^

**The values are reported as % rel of the total fatty acid peak areas detected in the GC analysis. They are mean values ± SD of the n repetitions of the same experiment.**

a FAME are obtained from total lipid extraction, derivatization, and GC analysis.

b The identification of the peaks have been performed by authentic samples and the identified peaks accounted for >98% of the total peaks.

c Values higher than untreated control (***P = 0.0001).

d Values lower than untreated control (***P = 0.0001).

e Values higher than untreated control (**P<0.001).

f Values lower than untreated control (**P<0.001).

g Values higher than untreated control (*P<0.01).

h Values lower than untreated control (*P<0.01).

* Evaluated with standard compounds (mono-trans arachidonic acid isomers) obtained following references [12].

# This value includes EPA and DHA.

**Table 3:** Membrane phospholipid fatty acids of NB100 cells treated for the indicated times with 150 µM PA, 50 µM OA, 50 µM AA and compared to controls grown in the same conditions without fatty acid supplementation.

FAME^a^	Control^b^	30′^b^	1 h^b^	1 h30′^b^	2 h^b^	2 h30′^b^	3 h^b^
	(n = 6)	(n = 4)	(n = 4)	(n = 4)	(n = 4)	(n = 4)	(n = 6)
16∶0	25.5±1.3	29.0±3.3	32.1±0.3 c	33.3±0.9 c	33.1±3.0^c^	29.8±3.1	37.1±2.6^c^
9*c*-16∶1	5.6±0.1	3.9±0.9^d^	4.4±0.2 d	4.2±0.3 d	3.9±0.1^d^	4.0±0.0^d^	3.9±0.2^d^
18∶0	14.4±0.8	13.3±0.6	12.1±0.6^d^	10.9±0.2^d^	9.3±0.6^d^	8.7±0.6^d^	8.6±0.1^d^
9*t-*18∶1	0.2±0.0	0.2±0.0	0.2±0.0	0.2±0.0	0.2±0.0	0.2±0.0	0.2±0.0
9*c*-18∶1	32.5±0.1	29.6±1.5^d^	28.1±0.4^d^	27.3±0.8^d^	28.0±1.4^d^	29.1±1.4^d^	25.8±1.0^d^
11*c*-18∶1	6.5±0.2	5.5±0.2^d^	4.9±0.1^d^	4.6±0.4^d^	4.3±0.1^d^	4.3±0.1^d^	3.8±0.2^d^
9*c,*12*c*-18∶2	3.9±0.5	3.2±0.2^f^	2.8±0.2^d^	3.7±1.3	3.4±0.2	3.2±0.0^d^	2.7±0.1^d^
8*c*,11*c*,14*c*-20∶3	1.0±0.1	0.7±0.1^f^	0.8±0.1^f^	0.7±0.1^f^	0.7±0.1^f^	0.8±0.1	0.7±0.1^f^
5*c*, 8*c,*11*c*,14*c*-20∶4	5.9±0.2	10.8±1.4^c^	11.6±0.5^c^	12.5±0.3^c^	14.1±1.4^c^	16.5±1.9^c^	14.8±0.8^c^
*trans*-20∶4*	0.1±0.0	0.1±0.0	0.1±0.0	0.1±0.0	0.1±0.0	0.1±0.0	0.1±0.0
SFA	39.9±0.4	42.2±2.7	44.2±0.3^c^	44.1±0.7^c^	42.4±3.6	38.5±3.7	45.7±2.5
MUFA	44.6±0.4	39.0±0.9^d^	37.3±0.7^d^	36.1±1.6^d^	36.2±1.6^d^	37.4±1.4^d^	33.5±1.4^d^
PUFA	15.1±0.1	18.4±1.7^c^	18.1±0.3^c^	19.4±0.6^c^	21.1±2.0^c^	23.8±2.3^c^	20.7±1.1^c^
SFA/MUFA	0.9±0.0	1.1±0.1^c^	1.2±0.0^c^	1.2±0.1^c^	1.2±0.1^c^	1.0±0.1	1.4±0.1^c^
Tot. Omega 6	10.8±0.1	14.7±1.3^c^	15.1±0.4^c^	16.9±0.9^c^	18.1±1.7^c^	19.9±2.0^c^	18.1±0.9^c^
Tot. Omega 3#	4.2±0.6	3.7±0.4	3.0±0.6	2.5±0.7^d^	2.5±0.5^d^	3.9±0.3	2.6±0.5^d^

**Controls are the mean of cells cultured from 0 to 3 hours in the absence of fatty acid supplementation. The values are reported as % rel of the total fatty acid peak areas detected in the GC analysis. They are mean values ± SD of the n repetitions of the same experiment.**

a FAME are obtained from total lipid extraction, derivatization, and GC analysis.

b The identification of the peaks have been performed by authentic samples and the identified peaks accounted for >98% of the total peaks.

c Values higher than untreated control (*P = 0.01).

d Values lower than untreated control (*P = 0.01).

e Values higher than untreated control (*P<0.04).

f Values lower than untreated control (*P<0.04).

* Evaluated with standard compounds (mono-trans arachidonic acid isomers) obtained following references [12].

# This value includes EPA and DHA.

### Cell Culture

NB100 cells, derived from a human primary neuroblastoma [30],were from a Department cell collection and were originally provided by the Laboratory of Pediatric Oncology of the University of Bologna. Cells were cultured at 37°C in humidified atmosphere at 5% CO_2_ in complete medium (RPMI 1640 supplemented with 10% heat-inactivated FCS, 2 mM L-Glutamine, 100 units/mL Penicillin, 0.1 mg/mL Streptomicin). Cultures were maintained in the log phase of growth with a viability >95%. Cells were checked for the absence of Mycoplasma infection.

To subculture or to seed cells for experiments, the medium was removed and the cell monolayer was washed with PBS Ca^2+^/Mg^2+^ free. After five minutes of incubation with Trypsin/EDTA (200 mg/L EDTA, 500 mg/L Trypsin; 1 mL per 25 cm^2^ flasks), cells were harvested and centrifuged at 500×g for 5 minutes at room temperature. The pellet was re-suspended in complete medium and the required number of cells were seeded in flasks or plates.

### FA Supplementation. Viability Tests

Cells were checked for viability and adjusted to 4×10^4^ cells/mL in complete medium, then 100 µL of cell suspension were seeded in a 96-well microtiter plate. After 24 hours, each FA was dissolved in ethanol, diluted in complete medium and immediately added to cells (final concentration of ethanol <1%) [12].

In continuous incubation experiments, cells were exposed to FA for times ranging from 2 to 48 hours. In pulse and chase experiments, cells were treated with PA at concentration ranging from 50 to 150 µM, for 1 or 2 hours, and then incubated in complete medium for a total time of 48 hours.

Viability was determined after the indicated times by adding 20 µL/well of CellTiter 96 Aqueous One Solution Cell Proliferation Assay. The absorbance at 490 nm was measured after a one hour incubation at 37°C.

To analyze the effect of FA supplementation on membrane fatty acids, 1.5×10^6^ cells were seeded in 25 cm^2^ flasks in 5 mL of complete medium. After 24 hours of incubation, medium supplemented with FA was added. Cells and membranes were collected at different times, ranging from 0.5 to 24 hours, and analysed for fatty acid composition as described above.

### Western Blot Analysis of cPLA_2_

Cells (3×10^6^/20 mL) were seeded in 75 cm^2^ flasks and, after 24 hours, medium supplemented with 150 µM PA was added. After different periods of incubation (15 to 180 min), cells were harvested with a cell scraper, collected by centrifugation at 300×*g* for 5 minutes and washed twice in PBS. Cell pellets were lysed with 100 µL of Cell Lytic-M (Sigma-Aldrich) supplemented with Protease Inhibitor Cocktail (1∶100), Phosphatase Inhibitor Cocktail 1 (1∶100) and sodium-orthovanadate (1∶500) (Sigma-Aldrich). After 45 min at 0°C and vortexing every 5 min, insoluble material (nuclear pellet plus membranes) was removed by centrifugation at 12,000×*g* for 20 min at 4°C. Protein supernatant (cell lysate) was collected and stored a −80°C. Protein content was quantified by spectrophotometer and 80 µg/lane of protein were separated by SDS-PAGE (10% gel) and blotted to Immobilon (polyvinylidene difluoride, PVDF) membrane (Millipore). Non-specific antibody binding sites were blocked by incubation with blocking buffer, TRIS buffered saline, 0.1% Tween 20 (TBS/T) with 5% w/v non-fat dry milk, for 1 hour at room temperature. After 5 washes with TBS/T, membranes were incubated overnight at 4°C with anti-phospho-cPLA_2_ (Ser 505) mAb (Cell Signaling Technology, Inc.; Beverly, MA) diluted in TBS/T with 5% bovine serum albumin, according to the manufacturer’s instructions. After 5 washes with TBS/T, membranes were incubated for 1 hour at room temperature with horseradish peroxidase–conjugated anti-rabbit antibody (Sigma-Aldrich) diluted in blocking buffer. After further 5 washes, proteins were detected by incubating the membrane with Immobilon Western detection reagent (Millipore). The anti-phospho-cPLA_2_ antibody was then stripped, 30 min in 25 mM glycine-HCl pH 2, 1% SDS (w/v), and, after blocking with non-fat milk, the membrane was reincubated with an antibody that recognises total cPLA_2_ (Cell Signaling) to account for equal loading. Proteins were detected as above. The relative levels of expression of different proteins were determined by the public domain software Image J.

### Caspases Activation

The activities of caspase-2, -3/7, -8 and -9 were assessed by the luminescent assays Caspase-Glo™ 2, Caspase-Glo™ 3/7, Caspase-Glo™ 8 and Caspase-Glo™ 9 (Promega). Cells were checked for viability and adjusted to 6×10^4^ cells/mL in complete medium, then 100 µL/well of cell suspension were placed in a 96-well microtiter plate. After 24 hours, each FA was dissolved in ethanol, diluted in complete medium and immediately added to cells (final concentration of ethanol <1%). After incubation for the indicated time, cells were checked for viability. Caspase-Glo™ 2, Caspase-Glo™ 3/7, Caspase-Glo™ 8 or Caspase-Glo™ 9 reagent (50 µL/well) was added and the luminescence was measured by Fluoroskan Ascent FL (Labsystem, Helsinki, Finland) according to the manufacturer’s instructions. Luminescence values were normalised to cell viability.

Cell viability and caspase 3/7 were also evaluated at 24 hours in NB100 cells pretreated with the irreversible tetrapeptide pan-caspase inhibitor Z-VAD-fmk (30 µM) (Promega), added to cells 3 hours before FA supplementation.

### Statistical Analysis

Results are given as means ± SD. Statistical comparisons between FA values in Tables 1, 2, 3 were conducted using the SPSS software, version 13.0 (Chicago, IL), using t-test for group comparisons. Correlation coefficients were determined using Pearson’s test. Satistical significance was based on 95% confidence limits (p≤0.05).

Statistical analyses of data from cell viability, caspase experiments and Western blot analysis were conducted using the XLSTAT-Pro software, version 6.1.9 (Addinsoft, 2003) using a 95% confidence interval. Data were analyzed by ANOVA and ANCOVA with Bonferroni’s correction.

## Results

### Palmitic Acid Supplementation

Palmitic acid (PA)-induced apoptotic effects have been reported in several studies which lack of information on lipidome changes [16]–[21]. PA is an important dietary saturated fatty acid, which at concentrations <100 µM has no detrimental effects on cell viability [31]. We, therefore, were interested in analyzing potential membrane changes with the supplementation of this fatty acid below and above this level. In particular, we used different PA concentrations (50,75,100,125 and 150 µM) with neuroblastoma cells (NB100).

**Figure 1:**
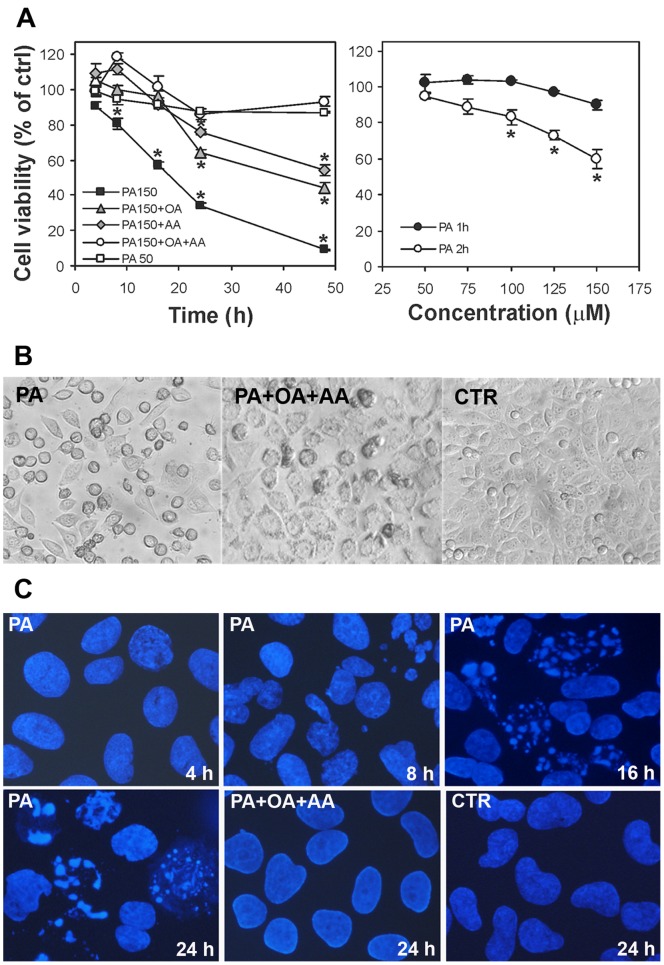
Effect of PA on NB100 cell line and cell morphology. (**A**) Effect of FA supplementation on NB100 cell viability. Cell viability was determined by MTS assay. Values are means ± SD of four determinations. Left graph: cells were incubated in complete medium supplemented with 50 µM PA (□), 150 µM PA (▪), 150 µM PA +50 µM OA (♦), 150 µM PA +50 µM AA (▴), 150 µM PA +50 µM OA +50 µM AA (○). Right graph: cells were incubated in complete medium supplemented with PA at various concentrations for 1 hour (•) or for 2 hours (○), and then incubated in complete medium for 48 hours after wash. (**B**) NB100 cells morphology assessed by phase contrast microscopy. Control cultures grown in the absence of FA supplementation are shown in comparison with cells treated for 24 hours with 150 µM PA or 150 µM PA +50 µM OA +50 µM AA. Magnification 200×. (**C**) Nuclei of NB100 cells stained with DAPI and assessed by fluorescence microscopy (×600 magnification objective). Cells were incubated in complete medium supplemented with 150 µM PA (4, 8, 16, 24 hours) or 150 µM PA +50 µM OA +50 µM AA (24 hours). Control cultures grown in the absence of FA supplementation (24 hours) are also shown.

Figure 1A highlights the results of the NB100 cell viability assay, in which the left graph shows the viability of NB100 cells in the presence of 50 and 150 µM PA under a continuous incubation up to 50 hours. PA at 150 µM dramatically reduced cell viability. Cell death was statistically higher than control cells already after 8 hours and was as high as 75% after 48 hours. On the other hand, PA at concentration of 50 µM caused marginally and non-significantly reduced cell viability. Pulse and chase experiments were then performed to analyze the fate of cells exposed for a short time (1–2 hours) to increasing concentrations of PA (50,75,100,125 and 150 µM), and then incubated in PA-free medium for additional 48 hours. The results are shown in Figure 1A (right graph). It is worth noting that a 2 hours incubation period, but not 1 hour, with 150 µM PA produces a clear effect on cell viability. Morphology of PA-treated cells was assessed by phase contrast microscopy. Cell morphology after a 24 hours treatment with 150 µM PA is shown in Figure 1B, in comparison with control cultures. Reduction of cell viability and apoptotic changes are clearly visible in cells treated with 150 µM PA.

Apoptosis was then monitored by fluorescence microscopy using DAPI nuclear staining. Figure 1C shows the nuclear morphology of control cells compared with cells treated for different times with 150 µM PA. Under this PA concentration, early apoptosis processes started at 8 hours with nuclear blebs eterochromatin organized in fine clumps. The apoptotic cells are characterized by compacting and margination of nuclear chromatin. After 16 and 24 hours, apoptotic cells in early karyorrhexis containing numerous micronucleations and late apoptotic cells characterized by peripheral nuclear fragmentation were also visible. In case of 50 µM PA, no such apoptotic features were detectable even at 24 hours of incubation (data not shown).

In parallel, the membrane fatty acid composition was monitored. Table 1 shows the follow-up every 30 minutes following the addition of 50 and 150 µM PA up to a three-hour incubation period, whereas Table 2 shows the follow-up of the fatty acid compositions at longer incubation times (8 and 24 hours), corresponding to the times of cell viability checkup shown in Figure 1. Two different PA concentrations (50 and 150 µM) are displayed in Table 2.

Table 1 shows that the PA incorporation in membrane phospholipids after 150 µM supplementation started already after 30 minutes of incubation and increased further along the time. Changes were statistically evaluated in comparison with cells without PA supplementation at the same incubation times. Since control cells did not change their membrane fatty acid composition along 3 hours incubation, in Table 1 the controls include the mean values of samples from 0 to 3 hours incubation (n = 9). Several changes in membrane phospholipids were associated to the progressive and significant incorporation of PA along incubation time,: *i*) palmitoleic acid (9cis-16∶1) temporarily decreased (from 1 to 2.5 hours) in a significant manner compared to controls (P<0.001). *ii*) stearic acid (18∶0) significantly diminished from 30 to 180 min of incubation (30–150 min, P<0.001; 180 min, P = 0.0001). The levels of monounsaturated fatty acids, oleic (9cis-18∶1) and vaccenic acids (11cis-18∶1), also diminished significantly (60–150 min, P<0.001; 180 min, P = 0.0001). *iii*) The level of arachidonic acid (20∶4) increased significantly at 30 min of incubation compared to controls (P<0.001), then started to diminish as the incubation progressed, reaching a nadir at 150–180. (P<0.001), P = 0.0001, respectively). Interestingly, the levels of the precursors of arachidonic acid (20∶4) in the omega-6 pathway, i.e., linoleic (18∶2) and eicosatrienoic (20∶3) acids, increased significantly during incubation, with a peak at 150 min, then lowering to control levels at 180 min (see Table 1). The whole omega-6 fatty acid family increased significantly at 3 hours incubation (P = 0.0001); *iv)* the omega-3 diminished during the different incubation times, reaching a value significantly lower than controls only after 3 h (P<0.001) (Table 1). At least for the first hours PUFA oxidative damage can be excluded, because this should have indiscriminately caused diminution of all polyunsaturated members (*cfr.,* 18∶2, 20∶3 and 20∶4 in Table 1) [32], [33].

The follow-up of the fatty acid compositions at 8 and 24 hours incubation for two different PA concentrations (50 and 150 µM) shows that the membrane fatty acid composition varies significantly compared to controls (Table 2). The extent of these changes is accentuated with 150 µM PA. In particular, differences between the two different concentrations can be observed with the values of SFA/MUFA ratio≥1.5, total omega-6≤5.4% and total omega-3≤1.9% that are reached only with 150 µM PA. These data, in parallel with the above reported cell viability and apoptotic changes (Figure 1), after 150 µM PA supplementation, indicate the crucial modifications of fatty acid balance in membrane phospholipids. It is worth recalling that cells naturally rich in omega-3 PUFA, such as nervous cells [34], take these essential fatty acids from fetal calf serum present in the medium. This could influence membrane lipidomics, however in the present experiments we followed the standard protocols for cell culture. When the wash-out of palmitic acid is carried out after 1 hour incubation, lipidomics shows that the membrane fatty acid composition at 8 and 24 hours did not change respect to control cells, most likely by allowing the re-equilibration of the membrane status (data not shown).

Due to the phospholipid structure with two fatty acid tails, the diminution of one fatty acid level can be correlated with another fatty acid residue in membrane phospholipids. Noteworthy, the diminution of arachidonic acid (20∶4) at 3 hours of incubation correlated with the decreased level of stearic acid (18∶0) (r = 0.987; p<0.05).

In our chromatographic conditions we were able to check the presence of trans fatty acids, monitoring the trans isomer of oleic acid (9*t*-18∶1) and the geometrical mono-trans isomers of arachidonic acid (*trans*-20∶4) reported in Tables 1 and 2 [12]. These isomers have been associated to the production of free radical isomerising species during cellular stress in cell, animal and human models [12]–[14], [35], [36]. In NB100 cultures exposed to PA, trans fatty acids diminished indicating that the free radical stress and geometrical transformation of the natural cis content do not associate directly with the effects of lipid remodelling.

**Figure 2:**
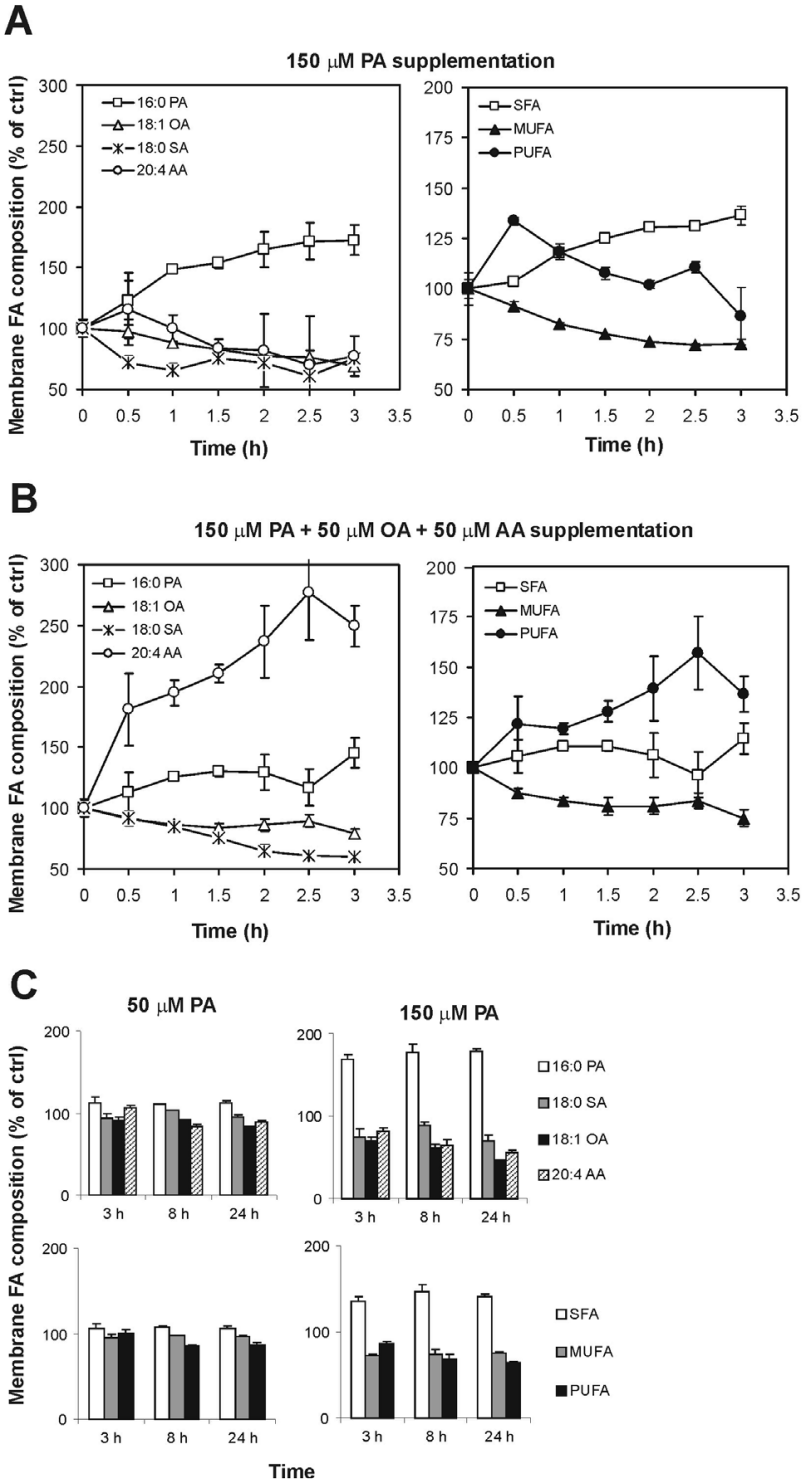
Main fatty acid changes in NB100 cell membranes after fatty acids supplementation. (**A**) In the left panel, the main fatty acid variations in NB100 cell membranes after 150 µM PA supplementation are reported. In the right panel, fluctuations of the corresponding fatty acid families are evidenced. (**B**) In the left panel, the main fatty acid variations in NB100 cell membranes after 150 µM PA +50 µM OA +50 µM AA supplementation are reported. The right panel evidences the fatty acid family changes after this supplementation. (**C**) In the left panel, the main fatty acid variations and the corresponding fatty acid families are evidenced in NB100 cell membranes after 50 µM PA supplementation. In the right panel, the same fatty acid variations are reported for cells incubated with 150 µM PA for 8 hours and 24 hours. Data are obtained from Tables 1, 2 and 3. Values are means ± SD. Statistical significances are as reported in the notes to the tables.

In Figure 2A the graph summarizes the main changes of 150 µM PA on the composition of NB100 membrane fatty acids throughout 3 hours exposures. Of importance is the 40% decrease in stearic acid (18∶0) content just after 30 minutes, whereas the content of arachidonic acid (20∶4) started to decrease after 90 minutes and was reduced by 30% at 3 hours.

### Oleic Acid and Arachidonic Acid Attenuate the Effects of PA Supplementation

The lipidome monitoring during palmitic acid supplementation highlighted a crucial role of arachidonic acid in NB100 cell cultures. In fact, the major effect in the membrane exposed to PA was the incorporation of this FA to the membrane concomitant with a significant loss in arachidonic acid. At this point it is worth mentioning that arachidonic acid plays crucial roles, either as bioactive molecule at a physiological concentration around 50 µM [37], and as fundamental component of the membrane lipidome of nervous cells, contributing to the lipid assembly and functioning [38]. It is worth recalling that the role of arachidonic acid can become even more crucial in the conditions of NB100 cells shorten of omega-3 fatty acids, as previously mentioned. On the basis of previously reported lipidome analyses, we envisaged that a concomitant addition of arachidonic (50 µM) and oleic (50 µM) acids could counterbalance the membrane fatty acid changes caused by 150 µM PA in the NB100 cultures. Figure 1A shows that the combined fatty acid supplementation rescued the cells from the lethal effect of palmitic acid. Figures 1B and 1C show that the co-supplementation maintained cell and nuclear morphology not different from the control cells at 24 hours incubation. Table 3 shows the analysis of membrane lipidome during this experiment and the graphs in Figure 2B summarize the behavior of specific fatty acids and families along the time. Under these conditions cell membranes were progressively enriched with palmitic acid, albeit not to the same extent as in the absence of arachidonic and oleic acids (Figure 2A), but yet at a significant manner (P = 0.01). This was accompanied with a significant decrease in the content of palmitoleic (16∶1; P = 0.01), stearic (18∶0, P = 0.01), oleic (18∶1 Δ9; P = 0.04), vaccenic (18∶1 Δ11; P<0.04), linoleic (18∶2; P<0.04) and eicosatrienoic acids (20∶3; P<0.04). Nevertheless, the cell uptake of arachidonic acid during the three hours incubation was significantly higher (P = 0.01). By comparing the data in Tables 1 and 3, it emerges a completely different effect of 150 µM PA in the absence or presence of oleic and arachidonic acids. The difference in the membrane fatty acid distribution seems to be effective for rescuing cells from apoptosis, focusing attention to the crucial role of PUFA levels in membrane phospholipids (Figure 2 A and B, right panels). In the case of PA, OA and AA combined supplementation we did not focus on the fatty acid analysis over long period (>3 hours) taking into account that: i) in the 150 µM PA supplementation changes were observed only at early times (within 3 hours, see Figure 2), ii) the combined supplementation gave a complete restoration of cell viability (see Figure 1A).

### cPLA_2_ and Caspase Activation

**Figure 3:**
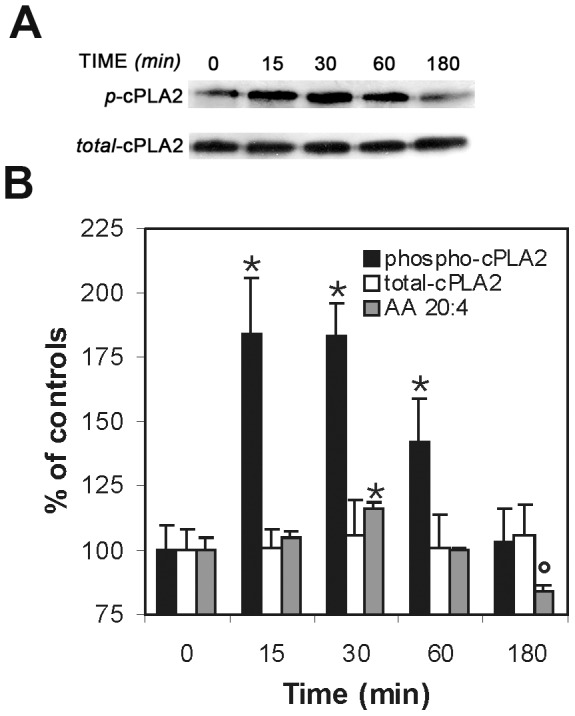
cPLA2 activation under PA supplementation. (**A**) Western blot analyses of the effects of PA treatment on cPLA_2_ protein activation in its phosphorylated form in NB100 cells. Cells were examined after treatment for 15–180 min with 150 µM PA. Cell lysates were resolved by SDS/PAGE. Proteins were blotted and detected with monoclonal antibodies against cPLA_2_ or phospho-cPLA_2_. Representative gels are shown. (**B**) The bar graph represents the band intensity values obtained by the Image J analysis, expressed as percentage of the corresponding control (Time 0) (white bars: total cPLA_2_; black bars: phospho-cPLA_2_). AA quantity in membranes as % of control (grey bars) is obtained from Table 1. Results are mean ± SD of at least three independent experiments. Phospho-cPLA_2_ is significantly higher than controls at 15, 30 and 60 min (p<0.0001). AA is significantly higher at 30 min and significantly lower at 180 min (p = 0.001).

The reduced content of arachidonic acid in membrane of cells treated with 150 µM arachidonic acid could result from the release of the former from phospholipids by PLA_2_. Indeed, PLA_2_ enzymes are known to be central regulators of stimulus-coupled cellular AA mobilization [39], [40], exhibiting a significant selectivity toward phospholipids bearing arachidonic acid moieties at the sn-2 position. Cytosolic PLA_2_ (cPLA_2_) activation can be monitored by a specific antibody detecting the phosphorylation at the Ser^505^ residue [41]. Figure 3 shows the Western blot analysis of cPLA_2_ in cells lysates following 150 µM PA supplementation. The total cell content of cPLA_2_ remained unaltered within the examined time (0–180 min). The cPLA_2_ activated form markedly increased 15 min after PA addition, remaining in plateau up to 30 min, and then decreased to the baseline at 180 min. Figure 3B depicts a summary of the Western blot analysis and combines it with the relative levels of archidonic acid in the cell membrane. These data shows a clear correlation of the arachidonic acid release from the cell membranes with the activation of PLA_2_ in the cells.

In order to further characterize cell death induction by the fatty acids, the effector caspases -2, -8 and -9 and the executioner caspase-3/7 were evaluated at different times (3, 8, 16 and 24 hours).

**Figure 4:**
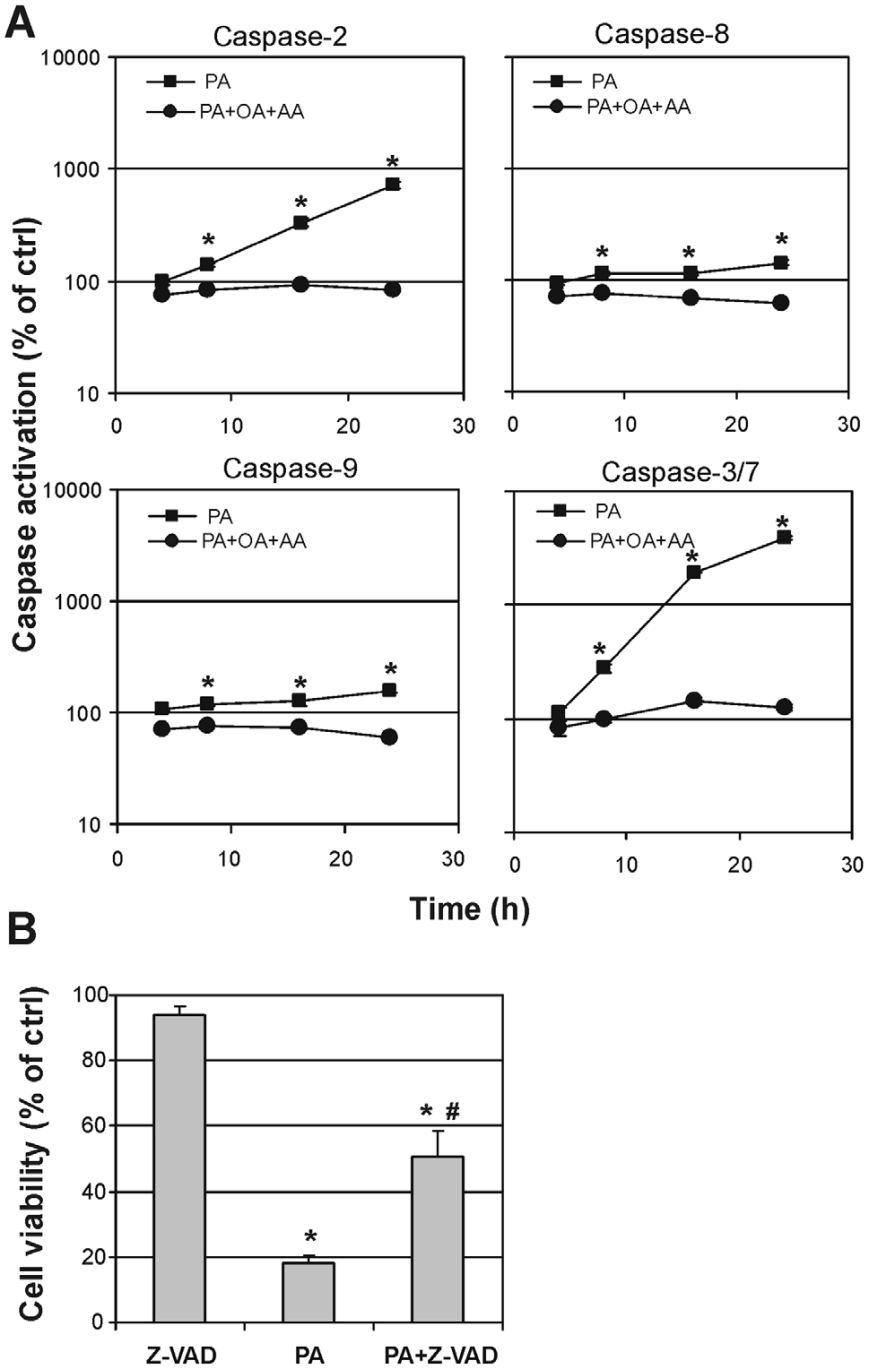
Evaluation of apoptosis in NB100 cells supplemented with fatty acids. (**A**) Caspase activation in NB100 cells exposed to PA 150 µM (▪) or PA 150 µM+OA 50 µM+AA 50 µM (•). Caspase-2, -8, -9 and -3/7 activation was determined at 3, 8, 16, 24 hours as described in Materials and Methods. Caspase activity is expressed as percentage of control values obtained form cultures grown in the absence of FA supplementation. Mean results ± SD are reported. (**B**) Cell viability was evaluated at 24 hours on NB100 cells pretreated with 30 µM of the irreversible tetrapeptide pan-caspase inhibitor Z-VAD-fmk, added to the culture 3 hours before the 150 µM PA supplementation.

The activity of each tested caspase increased linearly after incubation with palmitic acid, becoming highly significant starting from 8 h (p<0.0001) (Figure 4A). Particularly PA induced a strong activation of caspase-2 and 3/7, that augmented in exponential manner, reaching about 700 and 4000% of controls, respectively, after 24 hours of incubation. The simultaneous administration of OA and AA together to PA resulted in a complete lack of caspases activation (p<0.0001 by ANCOVA analysys with Bonferroni’s correction).

To evaluate the role of different mechanisms of cytotoxicity in PA-induced cell death we also used the pan-caspase inhibitor Z-VAD that is widely used as an anti-apoptotic agent. Z-VAD completely inhibited caspases in NB100 cells (data not shown) and reduced cell loss, yet, the inhibitor was only partially able to rescue cells from PA-induced death (Figure 4B).

## Discussion

The analysis of lipidome changes of neuroblastoma cell lines (NB100) was performed in the presence of increasing concentrations of palmitic acid (16∶0), a saturated fatty acid, which is important for cell growth [15]. Yet, at high levels this fatty acid it is also known for its apoptotic effects [16]–[21]. Particularly, the analysis included the levels of fatty acid residues and their natural geometry in membrane phospholipids. Change of the natural geometry of cis unsaturated fatty acid double bonds, with the formation of geometrical trans isomers, is an endogenous process that can be mediated by free radicals [10]–[13], as demonstrated in cell and animal models using a trans-free diet [12], [35]. The role of trans lipids in cell signalling of human metabolism has still to be defined [42], however recently trans lipids derived from dietary consumption have been correlated to brain conditions, in particular to aggressive character [43].

The disparate effect of PA, as a nutrient on one hand, and an inducer of apoptosis in neutral cells on the other, is intriguing [18]. The hypothesis that the release of free fatty acids during cerebral ischemia or other traumatic events can be toxic [16], [24], [25] is well correlated to the cytotoxic effects of PA. However, there is not yet an established link between the dietary supplementation of some fatty acids to the lipid composition and malfunction of cell membranes. By analyzing the membrane fatty acid composition, we found that palmitic acid supplementation increased the percentage of this fatty acid in NB100 membranes already within the first hour of incubation, along with the diminution of stearic acid (18∶0) and monounsaturated fatty acids (16∶1 and 18∶1), without affecting the overall polyunsaturated content (*cfr.*, Table 1). The highest dose of PA (150 µM) caused a pronounced loss of arachidonic acid (20∶4) after 1.5 hour of incubation, whereas linoleic (18∶2) and eicosatrienoic (20∶3) acids, the precursors of arachidonic acid, varied not uniformly and only at longer incubation times (cfr., Tables 1 and 2). The loss of PUFA components in the early hours of incubation seems not to be attributable to degradation by oxidative pathways, because these processes are unselective and affect the whole PUFA pattern [32], [33]. The evaluation of free radical stress levels was not performed specifically in this work and can be matter of further research.

The kinetics of cPLA_2_ activation in the PA-treated cells is consistent with a fast adaptation response to the palmitic acid supplementation, which occurs at the same time frame of the release of arachidonic acid from cell membrane phospholipids (cfr., Table 1 and Figure 3). The observation that the liberation of arachidonic acid matches with that of stearic acid, can even suggest that a specific phospholipid is released from membranes. In fact, as known from lipid biosynthesis, arachidonic acid is released from the sn-2 position of a phospholipid via the PLA_2_ activity, and the resulting lysophospholipid with a saturated fatty acid at the sn-1 position undergoes removal from membranes and turnover by replacement with new phospholipids [44]. Evaluating the results of lipidomic monitoring in Tables 1 and 2, and the changes reported in Figure 2, it is clear that lipid remodelling following palmitic acid supplementation at high concentration occurs at early times and the fatty acid changes are maintained at 8 and 24 hours, as shown in Table 2, thus affecting the cell signalling as demonstrated by the activation of caspases (in particular, caspases 3/7, 8, and 9, see Figure 4) after 8 hours. The careful early monitoring shown in Table 1 allowed profound changes at the level of signalling lipids (such as arachidonic acid) to be individuated promptly after supplementation of palmitic acid. This is the first time that an early monitoring of the fatty acid changes is provided during the examination of apoptotic cascade. This motivated the experiments of short-term exposure of the cells to PA. Exposure of the cells to 1 hour with the tested concentrations of palmitic acid (50–150 µM), followed by washing and further 48 hours incubation in PA-free medium, was inconsequential to the cells (Figure 1A, right). However, 2-hours incubation at high concentration (>100 µM) was sufficient to induce significant fatty acid changes (cfr., Table 1 and Figure 2A), which could not be avoided by simply washing and treating the cells with PA-free medium. The early changes can be appreciated in Figure 2A, where it is shown that cell membranes progressively incorporate saturated components (SFA) and loose unsaturated moieties (MUFA and PUFA) and this becomes crucial between 1 and 2 hours incubation. These experiments indicate that the fatty acid modifications at 1 hour PA incubation are still reversible by changing cell diet.

The effect of the combined supplementation of three fatty acids (palmitic, oleic and arachidonic acids) also enhances understanding of the role of fatty acids in maintaining cell function and integrity. The choice of oleic and arachidonic acids was based on their decrement observed during PA supplementation (Table 1 and Figure 2A, left panel). We chose 50 µM arachidonic acid as reported within the physiological range [37]. In the presence of PA, OA and AA, the PUFA phospholipid residues increase (cfr., Figure 2B and Table 3), likely to reduce the “stress” caused by the parallel incorporation of saturated fatty acids in membranes. A striking difference of the three fatty acid families can be appreciated by comparing the right panels of Figures 2A and 2B. This “diet” reduces the toxic effect of PA and rescues cells from apoptosis. This experiment also helps to exclude the hypothesis that lipoapoptosis could be involved when palmitic acid is supplemented and induces a fat overflow [19]. In fact, if the presence of high fatty acid concentrations is connected with apoptosis, the phenomenon could have occurred also when the fatty acid mixtures are used. Instead, it is clearly shown that the quality, more than the quantity, of the fatty acids is important for the apoptotic fate.

The high level of activation of caspase-3/7 and caspase-2 may result from a direct activation by ceramide, which are produced after membrane perturbation or a downstream activation, and the cleavage by caspase-3 during apoptosis [45]. However, the partial inhibition obtained by the pan-caspase inhibitor z-VAD-fmk indicates that caspase-independent pathways are involved in the cell response to palmitic acid supplementation (cfr., Figure 4). A caspase-independent cell death caused by free fatty acids has been already described in a neuronal cell model [18]. Arachidonic acid membrane release has been described and correlated with several apoptotic pathways. Arachidonic acid could trigger apoptosis directly by mitochondrial pathway or indirectly by the hydrolysis of sphingomyelin, which can produce ceramide [28]. However, the role of arachidonic acid in the present experimental system is intriguing, since it failed to induce significant apoptosis upon the combined supplementation. Recently, the effect of arachidonic acid supplementation in other neuronal cell lines (OLN-93) suggested it activates heat shock proteins (HSP-32), even at low concentrations (10 µM) [46]. These findings indicate that a broader scenario must be taken into consideration when the effects of supplementation and liberation of fatty acids in the medium and from cells are considered, in order to couple the membrane fatty acid information with other cellular factors, such as secretion of inflammatory mediators [47], [48], change of calcium levels, activation of enzymes and signalling cascades [17], [18], [21], [28], [29], [49]. This aspect is particularly important in other studies where palmitic acid supplementation in cell cultures is reported at concentration as high as 500 µM. Clearly, some of the assigned 'metabolic' effects of palmitic acid may result from the liberation of PUFA, and in particular archidonic acid, from cell membrane, as described in this study.

It is worth underlining that the effect of combined fatty acid supplementation cannot be completely understood by the present results and further work is needed to better clarify the mechanisms of protection afforded by OA/AA and to support the hypothesis of the central role of membrane remodeling.

In conclusion, membrane lipidome monitoring is a potent tool in cell biology experiments for the characterization of cells and the examination of membrane fatty acid reorganization. The results of this work give important molecular evidence that dietary fatty acids can mediate disparate effects, depending not only on the dose but even more importantly on the type of fatty acids and the exposure time used. Moreover, these results can inspire further work on the role of dietary fatty acids on the liberation pathways of free arachidonic acid from phospholipids in cells membrane and its role in inducing apoptosis.

## References

[1] van Meer G, Voelker DR, Feigenson GW (2008) Membranes lipids: where they are and how they behave. Nat Rev Mol Cell Biol 9: 112–124. 18216768 10.1038/nrm2330 PMC2642958

[2] Maxfield FR, Tabas I (2005) Role of cholesterol and lipid organization in disease. Nature 438: 612–621. 16319881 10.1038/nature04399

[3] Spener F, Lagarde M, Géloën A, Record M (2003) What is lipidomics? Eur J Lipid Sci Technol 105: 481–482.

[4] Watson AD (2006) Lipidomics: a global approach to lipid analysis in biological systems. J Lipid Res 47: 2101–2111. 16902246 10.1194/jlr.R600022-JLR200

[5] Wenk MR (2005) The emerging field of lipidomics. Nat Rev Drug Discov 4: 594–610. 16052242 10.1038/nrd1776

[6] Cevc G (1993) Phospholipids Handbook. Marcel Dekker ed., New York.

[7] Dowhan WR, Bogdanov M (2002) Functional roles of lipids in membranes. In: Vance JE, Vance DE, editors. Biochemistry of Lipids, Lipoproteins and Membranes, Elsevier, Amsterdam. 1–33.

[8] Engelman DM (2005) Membranes are more mosaic than fluid. Nature 438: 578–580. 16319876 10.1038/nature04394

[9] Christie WW (2007) The chromatographic analysis of lipids. In: Gunstone FD, Harwood JL, Dijkstra A, editors. The Lipid Handbook, Chapman and Hall, London. 426–455.

[10] Zhang YM, Rock CO (2008) Membrane lipid homeostasis in bacteria. Nat Rev Microbiol 6: 222–233. 18264115 10.1038/nrmicro1839

[11] Chatgilialoglu C, Ferreri C (2005) Trans Lipids: The Free Radical Path. Acc Chem Res 36: 441–448. 10.1021/ar0400847 15966710

[12] Ferreri C, Kratzsch S, Brede O, Marciniak B, Chatgilialoglu C (2005) Trans lipids formation induced by thiols in human monocytic leukemia cells. Free Radic Biol Med 38: 1180–1187. 15808415 10.1016/j.freeradbiomed.2004.12.026

[13] Ferreri C, Angelini F, Chatgilialoglu C, Dellonte S, Moschese V, et al (2005) Trans fatty acids and atopic eczema/dermatitis syndrome: the relationship with a free radical cis-trans isomerization of membrane lipids. Lipids 40: 661–667. 16196416 10.1007/s11745-005-1428-7

[14] Puca AA, Novelli V, Viviani C, Andrew P, Somalvico F, et al (2008) Lipid profile of erythrocyte membranes as possible biomarker of longevity. Rejuven Res 11: 63–72. 10.1089/rej.2007.0566 18160025

[15] Cook HW, McMaster CR (2002) Fatty acid desaturation and chain elongation in eukaryotes. In: Vance JE, Vance DE, editors. Biochemistry of Lipids, Lipoproteins and Membranes, Elsevier, Amsterdam. 181–202.

[16] Zhang JP, Sun GY (1995) Free fatty acids, neutral glycerides and phosphoglycerides in transient focal cerebral ischemia. J Neurochem 64: 1688–1695. 7891096 10.1046/j.1471-4159.1995.64041688.x

[17] Mu YM, Yanase T, Nishi Y, Tanaka A, Saito M, et al (2001) Saturated FFAs, palmitic acid and stearic acid, induce apoptosis in human granulosa cells. Endocrinology 142: 3590–3597. 11459807 10.1210/endo.142.8.8293

[18] Ulloth JE, Casiano CA, De Leon M (2003) Palmitic and stearic fatty acids induce caspase-dependent and -independent cell death in nerve growth factor differentiated PC12 cells. J Neurochem 84: 655–668. 12562510 10.1046/j.1471-4159.2003.01571.x PMC4157900

[19] Landau Z, Forti E, Alcaly M, Birk R (2006) Palmitate induced lipoapoptosis of exocrine pancreas AR42J cells. Apoptosis 11: 717–724. 16532273 10.1007/s10495-006-5425-3

[20] Kondoh Y, Kawada T, Urade R (2007) Activation of caspase 3 in HepG2 cells by elaidic acid (t18:1). Biochim Biophys Acta 1771: 500–505. 17321792 10.1016/j.bbalip.2007.01.012

[21] Yang X, Chan C (2007) Regulation of cellular apoptosis by palmitic acid and TNF-alpha: involvement of signal transduction pathways from PKR to Bcl-2. FASEB J 21: 943–924.

[22] Mangold HK, Malins DC (1960) Fractionation of fats, oils and waxes on thin layers of silicic acid. J Am Oil Chem Soc 37: 383–385.

[23] Abe K, Yoshidomi M, Kogure K (1991) Arachidonic acid metabolism in ischemic neuronal damage. Ann NY Acad Sci 559: 259–268. 10.1111/j.1749-6632.1989.tb22614.x 2774401

[24] Dhillon HS, Dose JM, Scheff SW, Prasad MR (1997) Time course of changes in lactate and free fatty acids after experimental brain injury and relationship to morphological damage. Exp Neurol 146: 240–249. 9225757 10.1006/exnr.1997.6524

[25] White BC, Sullivan JM, DeGarcia DJ, O’Neil BJ, Neumar RW, et al (2000) Brain ischemia and reperfusion: molecular mechanisms of neuronal injury. J Neurol Sci 179: 1–33. 11054482 10.1016/s0022-510x(00)00386-5

[26] Hammond EW (1993) Chromatography for the analysis of lipids. CRC Press, Cleveland. 188 p.

[27] Cao Y, Pearman AT, Zimmerman GA, McIntyre TM, Prescott SM (2000) Intracellular unesterified arachidonic acid signals apoptosis. Proc Natl Acad Sci USA 97: 11280–11285. 11005842 10.1073/pnas.200367597 PMC17191

[28] Penzo D, Petronilli V, Angelin A, Cusan C, Colonna R, et al (2004) Arachidonic acid released by phospholipase A(2) activation triggers Ca(2+)-dependent apoptosis through the mitochondrial pathway. J Biol Chem 279: 25219–25225. 15070903 10.1074/jbc.M310381200

[29] Matsuyama M, Yoshimura R, Mitsuhashi M, Tsuchida K, Takemoto Y, et al (2005) 5-Lipoxygenase inhibitors attenuate growth of human renal cell carcinoma and induce apoptosis through arachidonic acid pathway. Oncol Rep 14: 73–79. 15944770

[30] Sugimoto T, Tatsumi E, Kemshead JT, Helson L, Green AA, et al (1984) Determination of cell surface membrane antigens common to both human neuroblastoma and leukemia-lymphoma cell lines by a panel of 38 monoclonal antibodies. J Natl Cancer Inst 73: 51–57. 6610792

[31] Nano JL, Nobili C, Girard-Pipau F, Rampal P (2003) Effects of fatty acids on the growth of Caco-2 cells. Prostaglandins Leukot Essent Fatty Acids 69: 207–215. 12907129 10.1016/s0952-3278(03)00083-8

[32] Gutteridge JMC (1995) Lipid peroxidation and antioxidants as biomarkers of tissue damage. Clin Chem 41: 1219–1228. 7497639

[33] Girotti AW (1998) Lipid hydroperoxide generation, turnover, and effector action in biological systems. J Lipid Res 39: 1529–1542. 9717713

[34] Lauritzen L, Hansen HS, Jùrgensen MH, Michaelsen KF (2001) The essentiality of long chain n-3 fatty acids in relation to development and function of the brain and retina. Prog Lipid Res 40: 1–94. 11137568 10.1016/s0163-7827(00)00017-5

[35] Zambonin L, Ferreri C, Cabrini L, Prata C, Chatgilialoglu C, et al (2006) Occurrence of trans fatty acids in rats fed a trans-free diet: a free radical-mediated formation? Free Radic Biol Med 40: 1549–1556. 16632115 10.1016/j.freeradbiomed.2005.12.021

[36] Kermorvant-Duchemin E, Sennlaub F, Sirinyan M, Brault S, Andelfinger G, et al (2005) Trans-arachidonic acids generated during nitrative stress induce a thrombospondin-1-dependent microvascular degeneration. Nat Med 12: 1339–1345. 10.1038/nm1336 PMC4850227 16311602

[37] Brash AR (2001) Arachidonic acid as a bioactive molecule. J Clin Inv 107: 1339–1345. 10.1172/JCI13210 PMC209328 11390413

[38] du Bois TM, Deng C, Huang X-F (2005) Membrane phospholipid composition, alterations in neurotransmitter systems and schizophrenia. Progr Neuro-Psych Biol Psych 29: 878–888. 10.1016/j.pnpbp.2005.04.034 16005134

[39] Balsinde J, Winstea MV, Dennis EA (2002) Phospholipase A(2) regulation of arachidonic acid mobilization. FEBS Lett 531: 2–6. 12401193 10.1016/s0014-5793(02)03413-0

[40] Kudo I, Murakami M (2002) Phospholipase A2 enzymes. Prostaglandins Other Lipid Mediat 68–69: 3–58. 10.1016/s0090-6980(02)00020-5 12432908

[41] Leslie CC (1997) Properties and regulation of cytosolic phospholipase A2. J Biol Chem 272: 16709–16712. 9201969 10.1074/jbc.272.27.16709

[42] Mozaffarian D (2006) Trans fatty acids – Effects on systemic inflammation and endothelial function. Ather Suppl 7: 29–32. 10.1016/j.atherosclerosissup.2006.04.007 16713393

[43] Golomb BA, Evans MA, White HL, Dimsdale JE (2012) Trans Fat Consumption and Aggression. PLoS ONE 7(3): e32175 doi:10.1371/journal.pone.0032175 22403632 10.1371/journal.pone.0032175 PMC3293881

[44] Soupene E, Fyrst H, Kuypers FA (2008) Mammalian acyl-CoA:lysophosphatidylcholine acyltransferase enzymes. Proc Nat Acad Sci 105: 88–93. 18156367 10.1073/pnas.0709737104 PMC2224237

[45] Li H, Bergeron L, Cryns V, Pasternack MS, Zhu H, et al (1997) Activation of caspase-2 in apoptosis. J Biol Chem 272: 21010–21017. 9261102 10.1074/jbc.272.34.21010

[46] Brand A, Bauer NG, Hallott A, Goldbaum O, Ghebremeskel K, et al (2010) Membrane lipid modification by polyunsaturated fatty acids sensitizes oligodendroglial OLN-93 cells against oxidative stress and promotes up-regulation of heme oxygenase-1 (HSP32). J Neurochem 113: 465–476. 20096089 10.1111/j.1471-4159.2010.06611.x

[47] Andoh A, Takaya H, Araki T, Tsujikawa T, Fujiyama Y, et al (2000) Medium- and long-chain fatty acids differentially modulate interleukin-8 secretion in human fetal intestinal epithelial cells. J Nutr 130: 2636–2640. 11053499 10.1093/jn/130.11.2636

[48] Moghaddami N, Costabile M, Grover PK, Jersmann HP, Huang ZH, et al (2003) Unique effect of arachidonic acid on human neutrophil TNF receptor expression: up-regulation involving protein kinase C, extracellular signal regulated kinase, and phospholipase A_2_ . J Immunol 171: 2616–2624. 12928414 10.4049/jimmunol.171.5.2616

[49] McKenzie KE, Bandyopadhyay GK, Imagawa W, Sun K, Nandi S (1994) Omega-3 and omega-6 fatty acids and PGE2 stimulate the growth of normal but not tumor mouse mammary epithelial cells: evidence for alterations in the signaling pathways in tumor cells. Prostaglandins Leukot Essent Fatty Acids 51: 437–443. 7535935 10.1016/0952-3278(94)90062-0

